# Case Report: Presumptive subdural hemorrhage as a rare neurological manifestation of primary immune-mediated thrombocytopenia in a dog

**DOI:** 10.3389/fvets.2026.1969040

**Published:** 2026-10-14

**Authors:** Hsuan-Ping Hong, Tiffany G. Kei, Nicole A. Gagnon, Peter V. Scrivani

**Affiliations:** Department of Clinical Sciences, College of Veterinary Medicine, Cornell University, Ithaca, NY, United States

**Keywords:** canine, hematoma, immune-mediated thrombocytopenia, magnetic resonance imaging (MRI), seizure

## Abstract

A 10-year-old female spayed mixed-breed dog was presented with a history of seizures and acute progressive intracranial signs. The neurological examination was consistent with multifocal brain disease. Clinical examination revealed ecchymoses, petechiae, and bilateral retinal hemorrhage. Clinicopathological evaluation identified severe thrombocytopenia [a platelet count of 9 × 10^3^/μL without clumps on manual smear evaluation] with an increased mean platelet volume. Coagulation testing (activated partial thromboplastin time and prothrombin time) and fibrinolysis markers (D-dimers, quantitative fibrinogen), infectious disease screening, and thoracic and abdominal imaging were within normal limits, establishing a diagnosis of primary immune-mediated thrombocytopenia (ITP). Magnetic resonance imaging (MRI) of the brain showed minimally expanded, bilateral crescent-shaped, extra-axial fluid accumulations overlying both cerebral hemispheres. Based on T1-weighted and T2-weighted hyperintensities and peripheral susceptibility artifact on T2*-weighted gradient-recalled echo (GRE) imaging, a late subacute subdural intracranial hemorrhage was diagnosed. Despite initial uneventful anesthesia and recovery, the dog experienced acute neurological deterioration and suffered cardiopulmonary arrest, suspected due to ongoing hemorrhage or brain herniation. This report describes a rare neurological manifestation of primary ITP in a dog. While canine ITP generally carries a favorable prognosis, life-threatening secondary intracranial hemorrhage can occur. Primary ITP should be considered as a differential diagnosis for an acute, progressive neurological presentation until a normal platelet count is confirmed.

## Introduction

Hemorrhagic encephalopathies in veterinary medicine can be classified based on etiology or localization. A recently proposed veterinary classification scheme categorized hemorrhagic encephalopathy as primary, secondary, or traumatic, with subcategories for the well-used VITAMIN D scheme (1). Intracranial hemorrhage can be classified based on localization as extra-parenchymal, epidural, subdural, subarachnoid, subependymal, intraventricular, or intraparenchymal hemorrhage. In small animal veterinary medicine, intra-parenchymal and extra-parenchymal hemorrhages are well documented, whereas subdural hemorrhages or hematomas are rare and significantly underreported, particularly compared to human medicine. In dogs, subdural hemorrhages are rarely reported in traumatic brain injury cases, and their occurrence alongside conditions such as hydrocephalus and necrotizing encephalitis is rare, limited only to isolated case reports (2–4). Clinical signs associated with hemorrhagic encephalopathies vary considerably and depend predominantly on the acute or chronic stage of the hemorrhage, as well as the location, extent, secondary changes, and the overall underlying etiology.

Immune-mediated thrombocytopenia (ITP) is one of the most common hemostatic disorders in dogs (5). It can be either primary or secondary. Spontaneous hemorrhage typically does not occur until a platelet count of less than 30,000/μL. Clinical presentation of primary ITP is highly variable, with no specific correlation between the severity of the thrombocytopenia and the presenting clinical signs. Signs may include lethargy, inappetence or anorexia, petechiae and ecchymosis, and pyrexia. Less frequent manifestations include melena, hematuria, scleral hemorrhage, epistaxis, and hyphema, which can potentially be life-threatening (6). Several studies have reported variable short- and long-term outcomes of ITP in dogs, with an overall fair prognosis for survival to discharge. However, variable morbidity is associated with ITP in dogs, and the mortality rate can be up to 27% (7–9).

While typical mucosal and cutaneous hemorrhagic manifestations are well documented, neurological manifestations due to intracranial hemorrhage in ITP are uncommon to exceptionally rare in both the human and veterinary literature (10). This report describes a subdural hemorrhage as a rare and fatal neurological manifestation of primary ITP in a dog, highlighting the diagnostic challenges and clinical management within veterinary neurology.

## Case description

A 10-year-old female spayed mixed-breed dog was presented with an acute onset of an inability to stand and a history of seizure-like episodes. Over the past month, the dog was reported to have decreased activity, reluctance to jump, intermittent thoracic limb lameness and an acute onset of progressive seizure-like activity over the preceding 7 days. These episodes were characterized by whole-body rigidity with non-responsive mentation lasting 15–20 min that occurred a few times daily. Prior to referral, the dog was evaluated at an emergency clinic and subsequently at the primary care clinic, where a complete blood count, serum chemistry panel, and IDEXX 4Dx Plus SNAP® test were performed. Hematological evaluation revealed a mild leukocytosis (white blood cell count (WBC) 18.4 × 10^3^/μL; reference interval (RI) 4.0–15.5 × 10^3^/μL) and a severe thrombocytopenia (platelet count 21 × 10^3^/μL; RI 170–400 × 10^3^/μL) with a few small platelet clumps and an estimated decreased platelet count on blood smear. Biochemical analysis showed a mild hyperglobulinemia (3.7 g/dL; RI 1.6–3.6 g/dL) and a mild increase in alkaline phosphatase (138 IU/L; RI 5–131 IU/L). Electrolyte levels were within normal limits. The dog was negative on the 4Dx SNAP test for *Dirofilaria immitis* antigen and *Borrelia burgdorferi*, *Anaplasma* spp., and *Ehrlichia* spp. antibodies. The primary veterinarian prescribed an oral non-steroidal anti-inflammatory drug (NSAID; meloxicam) for suspected osteoarthritis and recommended referral if no improvement was seen.

On presentation, the dog was quiet, alert, and responsive. The physical examination was largely unremarkable except for an estimated 5% dehydration status. During the physical examination, the dog had another seizure-like episode, characterized by whole-body stiffness, falling over, and mydriasis with absent pupillary light reflexes in both eyes. The blood pressure during this episode was mildly increased (systolic, 150 mmHg; diastolic, 109 mmHg; mean arterial pressure [MAP], 113 mmHg). The neurological examination after the episodes showed a dull to obtunded mentation, absent proprioception in the right thoracic and pelvic limbs, and episodes of stiff thoracic limbs and neck extension. The dog was given a single dose of mannitol 0.5 g/kg intravenously (IV), maropitant 1 mg/kg IV, and was started on Plasma-Lyte at 60 mL/kg/day IV for overnight stabilization until transfer to the neurology service the next day. Overnight, the dog had an episode of neck extension, ptyalism, and increased respiratory effort that resolved without intervention; thoracic and abdominal point-of-care ultrasound (POCUS) was performed and did not show any B-lines or pleural, pericardial, or peritoneal effusion.

On the day of transfer to the neurology service, the dog was mildly to moderately dull with a low head and neck carriage. Due to the overnight seizure-like episodes, levetiracetam 32 mg/kg IV every 8 h was also added to the treatment. Physical examination revealed two focal ecchymoses on the left thoracic region, approximately 1.5 cm in length. Neurological examination showed mild ambulatory tetraparesis with vestibular ataxia, a tendency for a left head turn, mild left head tilt, and a single self-limiting episode of truncal titubation. On cranial nerve examination, the dog had absent menace response bilaterally, isocoric pupils with moderate to severe bilateral mydriasis, intact direct and indirect pupillary light reflexes bilaterally, and positional nystagmus shifting from vertical to rotary, with varying fast phase to the left or right. The dog had normal placing and hopping in the left thoracic and pelvic limbs, and inconsistent mildly delayed paw placement and hopping response in the right thoracic and pelvic limbs. Fundic examination showed bilateral retinal hemorrhage, worse in the right eye. The rest of the neurological exam was unremarkable. The neuroanatomical localization was multifocal brain (left forebrain and brainstem). Differential diagnoses included inflammatory or infectious meningoencephalitis, intracranial neoplasia, or vascular disease. A complete blood count and biochemistry panel were performed. Hematological evaluation revealed a mild leukocytosis (white blood cell count (WBC) 19.2 × 10^3^/μL; RI 5.7–14.2 × 10^3^/μL), reticulocytosis (absolute reticulocyte count 125.2 × 10^3^/μL; RI 11–92 × 10^3^/μL) with a normal hematocrit (46%; RI 41–58%), and a severe macrothrombocytopenia (platelet count 9 × 10^3^/μL; RI 186–545 × 10^3^/μL; mean platelet volume (MPV) 26.8 fL; RI 8.4–14.1 fL) with no visible platelet clumps and a markedly decreased platelet count on blood smear. Biochemical analysis showed a mild increase in alkaline phosphatase (148 IU/L; RI 7–115 IU/L). Comprehensive coagulation profile, including activated partial thromboplastin time (aPTT), prothrombin time (PT), quantitative fibrinogen (Clauss method), antithrombin activity (anti-IIa chromogenic substrate method), and quantitative cross-linked fibrin degradation products (D-dimer), was within normal limits. Additional diagnostics included thoracic radiography, which was unremarkable; abdominal ultrasound showed a moderately diffusely enlarged and heterogeneous spleen, most likely representing a benign or reactive change such as lymphoid hyperplasia or extramedullary hematopoiesis. Brain magnetic resonance imaging (MRI) was pursued the same day to further characterize the neurological clinical signs.

MRI was performed under general anesthesia using a 1.5-T scanner (Vantage Orian, Canon Medical Systems USA, Inc., Tustin, CA) with the dog in dorsal recumbency and a knee coil. Transverse-plane sequences included T1-weighted, T2-weighted, T2*-weighted gradient-recalled echo (GRE), fluid-attenuated inversion recovery (FLAIR), diffusion-weighted imaging (DWI), T1-weighted magnetization-prepared rapid gradient echo (MPRAGE), and pre- and post-contrast T1-weighted images (including a fat-saturated post-contrast T1-weighted sequence and a post-contrast T1 MPRAGE). Sagittal and dorsal planes included T2-weighted and post-contrast T1-weighted sequences. Apparent diffusion coefficient (ADC) maps were generated from the DWI data. Gadodiamide (Omniscan, GE Healthcare, Chicago, IL) was administered intravenously at a dose of 0.1 mmol/kg (1.3 mL total).

During image analysis, minimally expanded, bilateral crescent-shaped, extra-axial fluid accumulations were observed overlying the entire dorsolateral convexity of both cerebral hemispheres and not extending along the medial and basal borders (Figure 1). The fluid collections were homogeneously hyperintense on T1-weighted, T2-weighted, and FLAIR images with peripheral susceptibility artifact on T2* GRE images. Contrast enhancement could not be discerned because the fluid collections were already T1 hyperintense prior to contrast medium administration. No additional site of abnormal contrast enhancement was detected. The cerebral hemispheres were mildly compressed by the fluid collections and mildly diffusely swollen without midline shift. The compressed and swollen cerebrum displaced the diencephalon and mesencephalon caudally with compression of the rostral margin of the cerebellum and caudoventral cerebellar displacement extending to, but not through, the foramen magnum. Transverse imaging at the level of the tentorium confirmed that this caudal displacement was restricted to the midline tectum, with the parahippocampal gyri and cerebral cortex remaining normally positioned without caudal transtentorial herniation. At the level of the C2 and C3 vertebrae, the spinal cord was mildly swollen with poorly defined, locally extensive, non-contrast-enhancing, T2-weighted hyperintensity (Figure 2). The findings were interpreted as mild, bilateral, late subacute, intracranial subdural hemorrhage with mild secondary spinal cord edema. Intracranial pressure was estimated to be high based on intracranial displacement of the brain and spinal cord edema. As no other signs of trauma were observed, subdural hemorrhage secondary to severe thrombocytopenia was prioritized. Cerebrospinal fluid (CSF) analysis was not performed due to the patient’s severe thrombocytopenic state and suspected high intracranial pressure.

**Figure 1:**
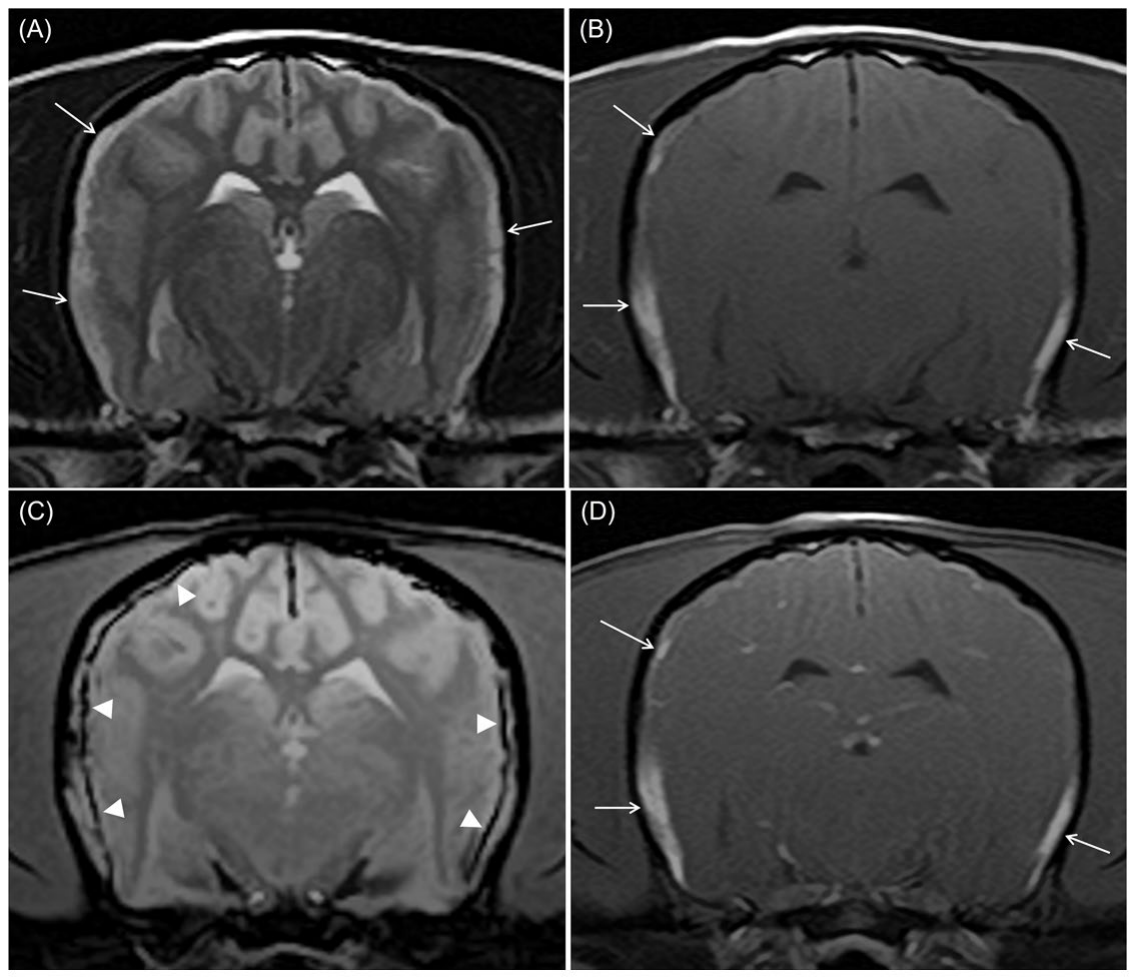
Transverse-plane canine brain magnetic resonance images demonstrating mild, bilateral, late subacute, subdural hemorrhage with mild cerebral compression and brain swelling; the right side of the patient is to the left of the images **(A–D)**. The subdural space is abnormally expanded with T2-weighted (T2W; **A**) and pre-contrast T1-weighted (T1W; **B**) hyperintense content (arrows). The T2*-weighted sequence **(C)** shows moderate peripheral susceptibility artifact overlying both cerebral convexities (arrowheads). The fat-saturated post-contrast T1-weighted image **(D)** shows no contrast enhancement of the same subdural content.

**Figure 2:**
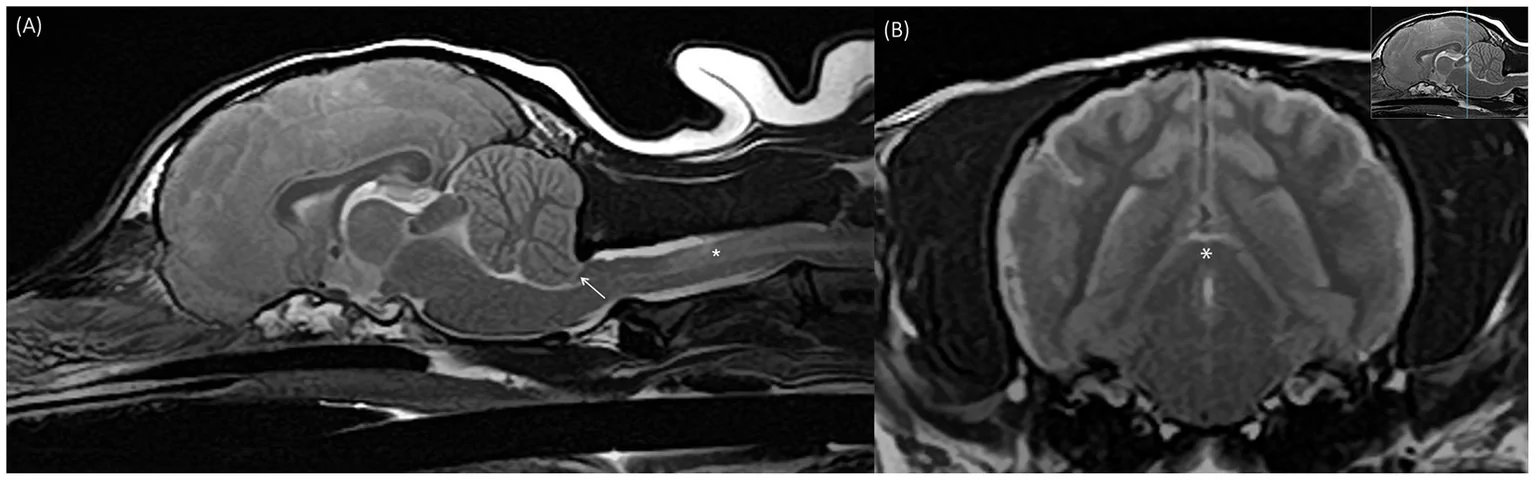
Magnetic resonance imaging (MRI) of the brain. **(A)** Sagittal-plane T2-weighted canine brain MRI image showing mild, diffuse brain swelling and caudal displacement of the mesencephalic tectum against the rostral margin of the cerebellum. Caudal displacement of the cerebellum extends to the level of the foramen magnum (arrow) without herniation. At the level of the C2 and C3 vertebrae, the spinal cord is mildly to moderately swollen over a locally extensive region, with T2-weighted (T2W) hyperintensity (*). **(B)** Transverse T2-weighted image at the level of the caudally displaced tectum (*, marked on the transverse image and sagittal inset). The slice passes through the tentorium cerebelli, allowing assessment of the adjacent parahippocampal gyri. The parahippocampal gyri are not displaced, indicating the absence of caudal transtentorial herniation.

Based on the clinical presentation, blood work, and imaging findings, the dog was diagnosed with a presumptive primary immune-mediated thrombocytopenia with secondary cerebral subdural hemorrhage. The dog was given a single dose of dexamethasone sodium phosphate of 0.1 mg/kg IV during recovery. The anesthesia and recovery were uneventful, and the dog was noted to be standing and walking in the intensive care kennel on its own with a quiet mentation. Three hours after recovery, on repeat neurological evaluation by the attending veterinarian, the dog was acutely and severely obtunded and exhibited moderate respiratory effort, though cardiopulmonary auscultation was unremarkable. This was subsequently followed by abrupt bradypnea, cyanosis of the tongue, and respiratory arrest, which rapidly progressed to cardiopulmonary arrest. Given the patient’s do-not-resuscitate (DNR) status, cardiopulmonary resuscitation (CPR) was not performed. The owner declined a postmortem examination.

## Discussion

This report describes a dog with multifocal neurological signs due to presumed bilateral intracranial subdural hemorrhages secondary to primary ITP, leading to a cardiorespiratory arrest. Only two individual case reports have previously described hemorrhagic myelopathies associated with presumed ITP in dogs (10, 11). In Guevar et al., the dog presented with an acute progressive ambulatory to non-ambulatory tetraparesis within 24 h, following a six-week history of thoracolumbar hyperesthesia and abnormal gait. The dog was diagnosed with presumed ITP based on a platelet count of 3 × 10^3^/μL and no other underlying cause. Cervical and thoracolumbar MRI revealed multifocal intradural-extramedullary and intramedullary hemorrhage within the thoracolumbosacral spinal cord. Despite the resolution of thrombocytopenia with immunomodulatory therapy, the dog’s neurological status deteriorated to tetraplegia with absent nociception in the pelvic limbs, and the dog was later euthanized. Postmortem examination showed multifocal intradural, subarachnoid, and intramedullary hemorrhage throughout the entire spinal cord with variable compression within the vertebral column. The second case describes a similar neurological presentation, where a dog with acute onset of tetraplegia with decreased sensation in all four limbs was diagnosed with presumed ITP. MRI of the cervical spine showed intramedullary hemorrhage. Immunomodulatory therapy was initiated with a combination of dexamethasone sodium phosphate and cyclosporine. The dog’s thrombocytopenia improved and resolved by day 5 after presentation, and the neurological status improved to non-ambulatory tetraparesis. At follow-up 5 weeks after discharge, the dog exhibited only mild generalized ataxia with no paresis present and maintained a normal platelet count.

These two cases highlight the potential severity of secondary neurological manifestations of ITP and emphasize the variability of clinical outcomes despite achieving normalized platelet counts. ITP in dogs is a complex disease characterized by a lack of direct correlation between the severity of thrombocytopenia and the anatomical location or clinical signs of the hemorrhage (12). While subclinical signs in dogs and cats are common even with severe thrombocytopenia, others may present with catastrophic, life-threatening hemorrhage. Studies of prognostic indicators have indicated that the presence of melena or high blood urea nitrogen (BUN) concentration may suggest a poor prognosis for affected dogs (8). However, no specific studies have evaluated whether the presence of neurological signs, either encephalopathies or myelopathies, poses a greater risk for recurrence or is associated with poor outcomes. In the case described here, neither melena nor increased BUN concentration was noted. Instead, the dog suffered sudden death following acute progression to obtundation, abrupt bradypnea, and cardiopulmonary arrest within 24 h of being diagnosed with ITP and presumed bilateral cerebral subdural hemorrhage. In the absence of a necropsy, a definitive postmortem cause of death could not be confirmed. Although pulmonary thromboembolism (PTE) is a recognized differential for sudden death in dogs, PTE typically presents with hyperventilation and tachypnea due to ventilation-perfusion mismatch and stimulation of the respiratory center. In contrast, the abrupt onset of bradypnea following obtundation in this patient strongly suggests brainstem dysfunction—specifically, suppression of the medullary respiratory center and ascending reticular activating system, potentially due to a rapid increase in intracranial pressure. At the time of MRI, mild diffuse cerebral swelling was evidenced by caudal displacement of the diencephalon and mesencephalon, along with compression of the rostral cerebellum and caudal displacement of the cerebellum toward the foramen magnum. Acute secondary events—such as dynamic expansion of the intracranial hemorrhage, progressive vasogenic edema, or increased intracranial pressure—are plausible contributors to the patient’s acute clinical deterioration; however, in the absence of necropsy confirmation, the definitive cause of death remains undetermined.

A primary limitation of this case is the absence of a postmortem examination, which precludes definitive pathological confirmation of the cerebral subdural hemorrhage and its direct relationship to the patient’s clinical deterioration and death. Furthermore, although immediate postmortem CSF analysis was feasible and could have aided in ruling out primary infectious or inflammatory meningoencephalitis or other etiologies, CSF findings following intracranial hemorrhage are often nonspecific, typically yielding variable cell populations and secondary erythrophagocytosis. Nonetheless, the combination of characteristic MRI features, comprehensive systemic diagnostics, and the patient’s clinical course provides antemortem evidence supporting a presumptive diagnosis of cerebral subdural hemorrhage as a rare neurological manifestation of primary immune-mediated thrombocytopenia.

Presently, ITP in dogs remains a diagnosis of exclusion (5). Due to its heterogeneous nature, standardizing the diagnostic approach and establishing definitive criteria have historically been challenging. Recently, the American College of Veterinary Internal Medicine (ACVIM) published an updated consensus statement on the diagnosis of ITP, providing a diagnostic algorithm for systemic exclusion of other underlying etiologies for thrombocytopenia. In the case described here, clinicopathological findings included severe thrombocytopenia (9 × 10^3^/μL) and increased mean platelet volume. Per the ACVIM consensus guideline, although dogs with ITP may have more severe thrombocytopenia than dogs with non-immune-mediated causes, clinical overlap persists. However, a platelet count cutoff < 20,000/μL strongly supports a diagnosis of ITP. A bone marrow examination was not performed in this case, and the current literature suggests there is insufficient evidence to determine whether bone marrow evaluation significantly improves the diagnostic accuracy of primary ITP in dogs. The consensus statement strongly recommends performing routine coagulation testing (aPTT, PT) and fibrinolysis markers (D-dimers, quantitative fibrinogen) during initial diagnostic workup to rule out concurrent consumption coagulopathies. In our case, these parameters were within normal limits. Comorbidity screening in ITP guidelines includes vector-borne infections; the dog tested negative for *Dirofilaria immitis* antigen and *Borrelia burgdorferi*, *Anaplasma* spp., and *Ehrlichia* spp. antibodies. Prior to referral, a dose of a non-steroidal anti-inflammatory drug (meloxicam) was administered. However, both the severe thrombocytopenia and clinical signs were present before this medication was given. Although the overall evidence for drug-induced ITP in dogs is limited, there is a high-to-intermediate level of evidence for cefazedone and sulfonamide drugs as triggers for ITP. However, the clinical timeline in this case most effectively excludes meloxicam as the primary inciting trigger.

Overall, the diagnostics performed in this case aligned with the ACVIM consensus statement for primary ITP. For dogs presenting with primary ITP and a platelet count < 50,000/μL, such as in this case, the consensus guideline suggests that there is moderate evidence that the initial admission platelet count does not impact disease outcome or therapeutic response. Rather, the severity of the platelet count may correlate predominantly with active clinical hemorrhage at the time of presentation, manifesting in this case as ecchymoses, bilateral retinal hemorrhage, and the intracranial subdural hemorrhage. Retinal hemorrhages are well documented as a severe ocular lesion in dogs with thrombocytopenia, with the lesion severity being associated with platelet count (13). Generally speaking, spontaneous bleeding typically occurs with a platelet count of < 30,000/μL; however, no prospective veterinary studies have compared long-term outcomes or site-specific fatality rates in dogs with primary ITP with mild (platelet count > 75,000/μL) versus moderate to severe (platelet count < 50,000/μL) thrombocytopenic states.

In the human literature, ITP is an uncommon hematological condition, and intracranial hemorrhage is considered a rare but fatal complication in severe thrombocytopenic patients, with an estimated mortality rate of 25–55% (14, 15). There is limited consensus regarding the optimal management of life-threatening intracranial hemorrhage, particularly for emergent surgical decompression to alleviate increased intracranial pressure during rapid deterioration of neurological signs. Only a single human case report has documented an excellent clinical outcome following a combination of emergency splenectomy and craniotomy (15). In our case, neurological deterioration and eventual cardiopulmonary arrest were rapid, despite an initial uneventful anesthesia and recovery. Given the lack of larger-scale prospective studies or established surgical guidelines for intracranial hemorrhagic events in veterinary patients, this case stands as the first case in the veterinary literature that demonstrates severe and fatal subdural hemorrhage as a primary neurological manifestation of ITP.

Although ITP has not been documented as a common etiology or differential for myelopathies or encephalopathies in dogs and cats, secondary central nervous system hemorrhage can occur due to a variety of underlying conditions. Secondary causes of hemorrhagic encephalopathies include vascular malformations, neoplasia, vasculitis associated with infectious or non-infectious meningoencephalitis, coagulopathies, ischemic infarcts with hemorrhagic transformations, metabolic or nutritional disorders, or heat stroke. Structurally, hemorrhage can be categorized as extra-parenchymal, epidural, subdural, subarachnoid, subependymal, intraventricular, and intraparenchymal based on location. While subdural hemorrhage is often clinically defined as blood accumulation within the space between the dura mater and arachnoid membrane, neuroanatomically it is recognized as an intradural lesion, as no true physiological subdural space exists. Rather, this pathologically expanded space is created when mechanical trauma or spontaneous vascular extravasation cleaves the structurally weak dural border cell layer, along with rupture of bridging vessels (1, 16). In the human literature, subdural hematoma most commonly occurs with traumatic brain injury, though it can occur in rare instances with vascular malformations and aneurysms; recognized risk factors include age, concurrent coagulopathies, anticoagulant therapy, brain atrophy, and alcohol disorders (17, 18). Conversely, in the veterinary literature, only a few subdural hemorrhages in dogs have been reported. Documented underlying etiologies include trauma, hydrocephalus, complications of lumbar myelography and ventriculoperitoneal shunt placement, and secondary venous tearing with brain atrophy due to ceroid lipofuscinosis (2, 3, 19–22). Typical neurological signs include seizures, altered mentation, and concurrent neurological deficits.

On MRI, cerebral subdural hematoma characteristically appears as single or multiple crescent-shaped extra-axial fluid collections with variable signal intensities dictated by the chronicity of hemorrhage. As blood products degrade and settle into gravity-dependent layers, the composition of the fluid accumulation changes over time, a unique phenomenon of fluid-filled spaces that does not occur in solid tissues. The MRI appearance of intracranial hemorrhage is highly complex, with signal intensity determined primarily by the T1 relaxation and T2* susceptibility effects of hemoglobin degradation and oxygenation over time. The subsequent degradation of hemoglobin, progressing from oxyhemoglobin to deoxyhemoglobin, intracellular methemoglobin, extracellular methemoglobin, and eventually ferritin and hemosiderin, alters the magnetic properties of the hematoma over time. These predictable biochemical variations allow the distinct stages of intracranial hemorrhage (hyperacute, acute, early subacute, late subacute, and chronic) to be determined on MRI (23).

Based on the MRI features in the present case, the dog’s cerebral subdural hemorrhage is most characteristic of the late subacute stage. This classification is supported by the T1-weighted and T2-weighted hyperintensity with susceptibility artifact on T2* GRE, and a characteristically expanded crescent-shaped subdural space surrounding both cerebral hemispheres. Crucially, although the diagnosis of ITP was established just prior to the MRI diagnosis of a subdural hemorrhage, the neurological signs, specifically seizure episodes, began approximately 1 week prior. This clinical timeline correlates closely with the late subacute to early chronic hemorrhage stage, which generally spans from days to months and further validates the severe thrombocytopenia and primary ITP as the etiology of this patient’s neurological signs.

## Conclusion

This case documents a rare clinical manifestation of ITP that has not previously been reported in the veterinary literature. ITP should be considered as a differential diagnosis for a dog presenting with acute progressive intracranial multifocal neurological deficits until an adequate platelet count is confirmed. Although ITP generally carries a favorable prognosis when managed appropriately, severe complications such as intracranial hemorrhage may result in significant morbidity and may be associated with a poor outcome.

## Data Availability

The original contributions presented in the study are included in the article/supplementary material; further inquiries can be directed to the corresponding author.
